# Development and Cross-Cultural Adaptation of the Tamil Version of the Stroke-Specific Quality of Life Scale (SSQoL) and Assessment of its Reliability and Validity

**DOI:** 10.7759/cureus.72404

**Published:** 2024-10-25

**Authors:** Shyam Sundar R, Deepak S, Vijaya Kumar K, Rajasekar S, Premkumar M

**Affiliations:** 1 Neurophysiotherapy, Srinivas University Institute of Physiotherapy, Mangalore, IND; 2 Neurosurgery, Srinivas Hospital, Mangalore, IND; 3 Physiotherapy, Kasturba Medical College, Manipal Academy of Higher Education, Mangalore, IND; 4 Musculoskeletal Physiotherapy, Srinivas University Institute of Physiotherapy, Mangalore, IND; 5 Cardiovascular and Pulmonary Physiotherapy, Srinivas University Institute of Physiotherapy, Mangalore, IND

**Keywords:** chronic stroke survivors, content validity, cross-cultural adaptation, stroke-specific quality of life scale, test-retest reliability

## Abstract

Background: In this current modern industrial world, strokes are the major reason for causing disability and death in the adult population. In spite of the various tools available to measure the physical, psychological, and social impact of strokes, the appropriate method in various languages around the world is not available. In that sense adapting the Stroke-Specific Quality of Life Scale (SSQoL) in different languages and cultures is essential to ensure their validity and efficacy across diverse populations.

Aim: This study aims to translate the original SSQoL English version into the Tamil language and assess the scale’s reliability and validity among Tamil-speaking subjects with chronic stroke survivors.

Methods: A methodological framework was applied to translate and culturally adapt SSQoL, involving forward and backward translation, committee review, and testing. A total of 220 participants were recruited to assess demographic characteristics, validity, and reliability of the Tamil-translated SSQoL-T using measures such as internal consistency, test-retest reliability, and convergent validity.

Results: The content validity analysis of the translated Tamil version of SSQoL-T showed strong positive outputs for both total score and sub-score assessments. In test-retest reliability analysis, good reliability with Cronbach’s alpha (≥0.9) was observed for both total score and sub-score assessments.

Conclusion: This study's findings underscore the content validity and good reliability of SSQoL-T as a screening tool for assessing stroke among Tamil-speaking populations, providing valuable insights for clinicians and researchers in the assessment and management of strokes.

## Introduction

Strokes play a vital role in adult disability and stand as the second reason for adult mortality after cardiac diseases in this advanced modernized world. Even though physical, social, and psychological factors of stroke impact the outcome of stroke, a suitable assessment instrument to analyze and measure outcomes of stroke is not available. As various advanced medicines have been analyzed for their effectiveness in clinical trials on stroke, outcomes related to stroke rehabilitation are also very essential, because of their relevancy and importance in the overall functional outcome of stroke patients [1].

All aspects of human life have been affected by any form of stroke, whether it is physical, intellectual, cognitive higher functioning, or psycho-social aspects of stroke patients and stroke survivors. Return to normal life and recovery in stroke patients are not homogeneous; more than 25% to 74% of stroke survivors out of 0.05 billion survivors of stroke condition throughout this world, need minimal assistance to fully dependent on attendants and relatives for their day-to-day life. In the past 40 years, there has been a two-fold increase in the occurrence of stroke in India like in moderate-income countries. At the same time as the advancement in overall healthcare, many stroke survivors have found it difficult to cope with all domains of their life including physical, psycho-social, and functional well-being [2-4].

In India, stroke stands at the fifth position as the reason for disability-adjusted life [5]. More than six million stroke cases happened in India, and that was the reason for more than 7% of total deaths in 2016 [6]. Ischemic changes lead to damage to the cerebral structures in stroke which may bring defects in cognitive, motor, and sensory functions [7], and survivors of stroke may require minimum short-duration support to maximum throughout life support, which leads to huge economic and human costs [8]. The ever-increasing geriatric population has been parallel with a significant rise in cardiovascular diseases and cerebrovascular disease fatality from 156 to 209 per 1 lakh from 1990 to 2017 [5]. It plays a vital role in the increased hospitalization (nearly 18.1%), and frequent hospital visits and consultations (nearly 32%) among the geriatric population in India for heart diseases and stroke, which was reported from the 75th Indian National Sample Survey [9].

For the past three decades, the assessment of the quality of life (QoL) of stroke patients has played a significant part in the overall analysis of outcomes in patients with stroke and their management. There is no general or universal definition for QoL. It has been accepted that QoL is always more than one-dimensional, with physical, mental, and social as its construct. The patient’s perception and understanding of their condition, and life, the effectiveness of management or intervention methods, and the perceived well-being of their life are the general parameters used by medical personnel and researchers related QoL concept medical field [10].

Some available instruments, questionnaires, and tools provide concepts about the subjective status of health like physical and psycho-social functioning, and to effectively do day-to-day activities or resistance to perform these functions [11]. Stroke-Specific Quality of Life Scale (SSQoL) questionnaire has been used as a universal tool to measure the physical, social, psychological, and functional outcomes of stroke survivors. The valid and reliable original SSQoL English version was used to assess the QoL in stroke survivors in this study. The SSQoL has overall 49 items and 12 areas of human life that are present in stroke survivors. Already SSQoL's original English version has been cross-culturally adapted, translated, and validated for use in many languages throughout the world [11-21].

Research objectives

Based on available published research, cross-cultural adaptation, translation, and validation have not been done or used in any of the South Indian languages. More than eight crore (80 million) people use Tamil language as their official working language throughout the world. The presence of a cross-culturally adapted, translated Tamil version of the SSQoL may help to increase the use of SSQoL-T among the researchers, medical personnel, and Tamil-speaking population.

## Materials and methods

**Figure 1:**
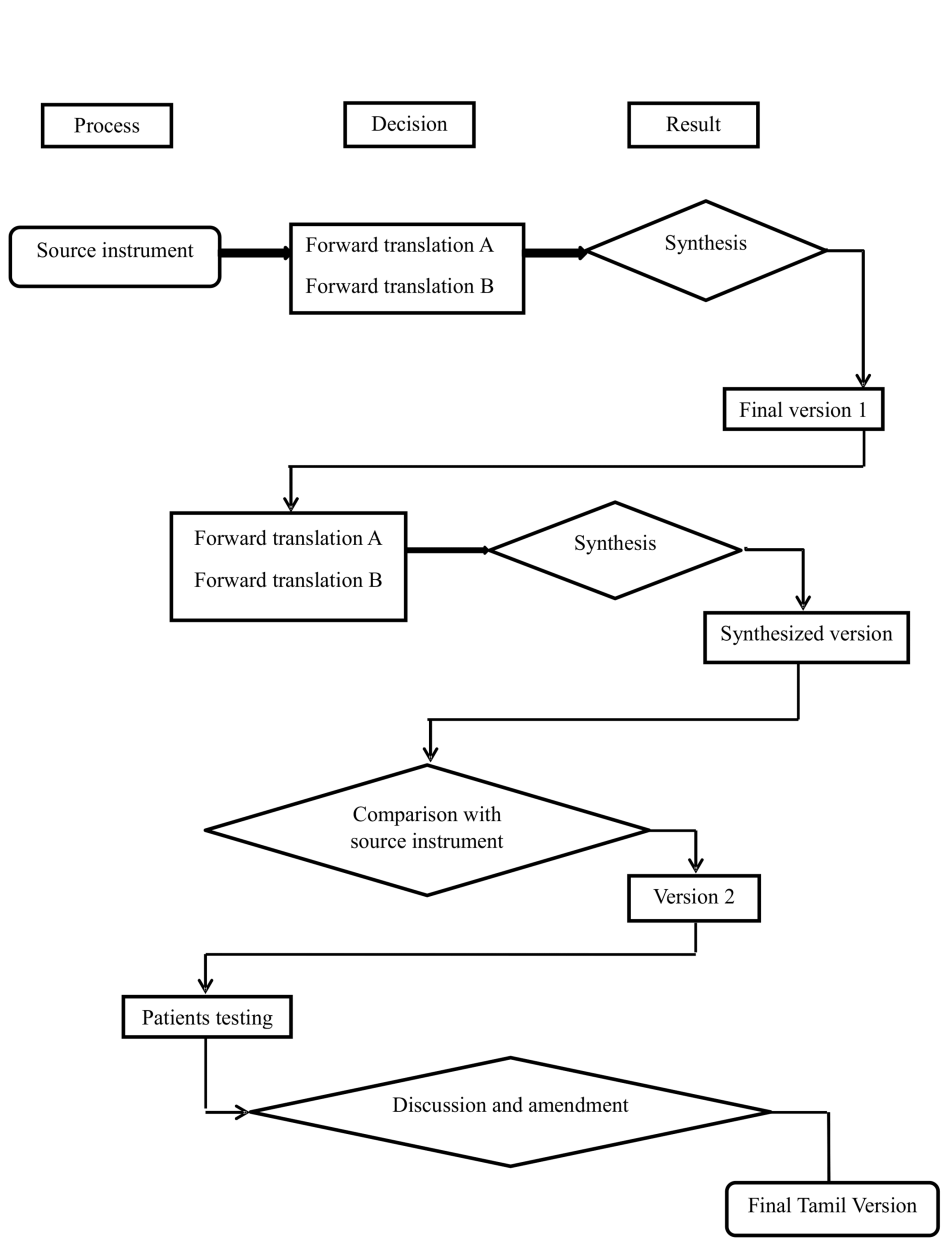
Steps in translation process

This study was conducted in two phases. In phase one, cross-cultural adaptation and translation of the original English version of SSQoL to the South Indian language Tamil (SSQoL-T). The psychometric properties of SSQoL-T were tested in phase two (Figure 1).

Ethical clearance was obtained from the Institutional Review Board of the Srinivas University Ethics Committee (Ref. No.: 17/Physiotherapy/2023). Subsequently, the investigator commenced by engaging participants experiencing stroke rehabilitation at centers in Madurai District, Tamil Nadu. From November 20, 2023, to May 20, 2024, based on selection criteria, 220 participants were recruited for this study. Informed written consent forms were taken from the study participants. The principles of the Helsinki Declaration were followed in this study.

Outcome measures were measured and documented by a qualified physiotherapist with a post-graduate degree who had three years of experience as a tester. Beaton guidelines of translation and cross-cultural adaptation were used as testing equipment to draw cultural adaptation, test-retest reliability analysis, and convergent validation for the translated version of the Tamil version of the SSQoL from the English version of the SSQoL and SSQoL-T [22].

Phase 1: cross-cultural adaptation

To ensure the quality of adaptation this study followed six essential steps recommended.

Step 1: Initial Translation/Forward Translation

The SSQoL-T was initially translated into Tamil from its original English version. The forward translation process entailed the participation of two translators proficient in both English and Tamil languages. The first translator, with a medical background, was familiar with the concept being measured, while the second translator, with a non-medical background, was unaware of the concept being measured.

Step 2: Synthesis

A meeting was conducted between the two language experts to obtain a consensus based on the translated version of SSQoL-T while keeping in mind the original SSQoL-T. The two translated versions were compared and analyzed until there was consensus regarding translation synthesis, resulting in the formation of the final synthesized version.

Step 3: Back Translation

The completed Tamil version underwent back translation into English by two different professional linguists who did not contribute to the initial forward translation phase. These translators were unaware of the notion explored in the questionnaire. From these two back-translated English versions, a final synthesized version was developed. This final synthesized English version was contrasted with the primary English version. Next, the final Tamil version of the SSQoL-T was reviewed by a bilingual team. The final translated Tamil version of SSQoL was cross-verified by four people comprising one physician, one Tamil lecturer, and two physiotherapists. Their goal was to evaluate the need for cross-cultural adaptation and refine the tool for application among Tamil-speaking patients. The final stage of adaptation involved testing the pre-final version as a process check.

Step 4: Reviewers Committee

Their objective was to evaluate the need for cross-cultural integration and refine it for use among Tamil-speaking patients who were fluent in the language. In the final stage of adaptation, emphasis was placed on achieving semantic, idiomatic, experimental, and conceptual equivalence with the original back-translated SSQoL. This process ensured that the pre-final version accurately reflected the intended meaning and context of the tool.

Step 5: Pretesting

Cross-cultural adaptation was conducted by implementing the pre-final version of the Tamil version of SSQoL-T on 220 patients with stroke. There was no significant difference between the primary rendition and the Tamil edition of the SSQoL-T, the pre-final and final indexes were matched. The primary objective was to evaluate whether the translated index was comprehensible and whether the vocabulary and expressions were relevant to Tamil culture. The SSQoL-T was administered twice, with a 48-hour interval, to estimate its test-retest reliability.

Step 6: Validation of Study

The Tamil version of SS-QoL-T was contrasted with the original English edition of SSQoL to validate this index.

## Results

The total sample size was 220, calculated by G Power test 3.1 version for Windows (Heinrich-Heine-Universität Düsseldorf, Düsseldorf, Germany) with a CFI (comparative fit index) value of more than 0.95 and an RMSEA (root mean square error of approximation) value of 0.05 [22]. Demographic data and SSQoL scores did not follow a normal distribution (Kolmogorov-Smirnov test). Demographic data were expressed in frequencies and percentages. The characteristics of subjects like age, sex, religion, literacy, etc., have been expressed in mean± SD and median (interquartile range) (Table 1).

**Table 1:** Socio-demographic and clinical characteristics of stroke survivors for psychometric testing (n = 220)

Characteristics	Chronic stroke survivors (N = 220)
	N (%)
Age (mean ± SD) in years	60.21 ± 13.0
Male	92 (67.6)
Female	44 (32.4)
Urban	98 (72.1)
Rural	38 (27.9)
Uneducated	44 (32.4)
Primary school	54 (39.7)
Secondary school	22 (16.2)
Tertiary (college & above)	16 (11.8)
Orthodox Christian	113 (83.1)
Muslim	21 (15.4)
Protestant	2 (1.5)
Catholic	0 (0.0)
Single	13 (9.6)
Married	96 (70.6)
Divorced	7 (5.1)
Widowed	20 (14.7)
Farmers	13 (9.6)
Employed	16 (11.8)
Retired	22 (16.2)
Business	23 (16.9)
Others	62 (45.6)
Hypertension	85 (62.5)
Diabetes mellitus	15 (11)
Heart problems	32 (23.5)
Others	4 (2.9)
Right	86 (63.2)
Left	50 (36.8)
Ischemic	94 (69.1)
Hemorrhagic	42 (30.9)
Stroke duration (month) mean ± SD	22.6 ± 12.7

Test-retest reliability analysis was done by calculating intraclass correlation coefficient (ICC) values with Cronbach’s alpha values (Table 2). For convergent validation, Spearman's rank correlation was used to test the convergent validity of SSQoL-T with SF-36 scores (Table 3). Data was analyzed with IBM SPSS Statistics for Windows, version 25 (IBM Corp., Armonk, NY, USA).

**Table 2:** Reliability of the Tamil version of the SSQoL-T, internal consistency, intraclass correlation coefficient, and item mean values (N = 220) ICC: intraclass correlation coefficient; CI: confidence interval; UE: upper extremity; SSQoL-T: Stroke-Specific Quality of Life Scale-Tamil

SSQoL domains (items)	Internal consistency Cronbach’s α	Item mean		ICC (95% CI)
		Minimum	Maximum	
Energy (3)	0.994	8.350	8.509	0.988 (0.984, 0.991)
Family (3)	0.992	8.441	8.591	0.984 (0.980, 0.988)
Language (5)	0.997	14.691	14.973	0.993 (0.991, 0.995)
Mobility (6)	0.992	16.323	16.600	0.984 (0.980, 0.988)
Mood (5)	0.992	13.168	13.464	0.984 (0.979, 0.988)
Personality (3)	0.996	8.341	8.441	0.993 (0.991, 0.995)
Self-care (5)	0.994	13.414	13.577	0.988 (0.984, 0.990)
Social role (5)	0.994	13.464	13.641	0.987 (0.983, 0.990)
Thinking (3)	0.995	8.527	8.659	0.989 (0.986, 0.992)
UE function (5)	0.991	13.645	13.886	0.982 (0.977, 0.987)
Vision (3)	0.992	8.509	8.595	0.984 (0.979, 0.987)
Work (3)	0.992	8.523	8.732	0.984 (0.980, 0.988)
Total score (49)	0.993	135.395	137.668	0.986 (0.982, 0.990)

**Table 3:** Spearman’s correlation of SSQoL-T with the subscales of SF-36 (N = 220) ** Correlation is significant at the 0.05 level (2-tailed); * Correlation is significant at the 0.05 level (2-tailed); SSQoL-T: Stroke-Specific Quality of Life Scale-Tamil r2 is coefficient of determination

SSQoL domain	Comparison of SF-36 scales	Spearman’s rho	r^2^	P
Energy	Vitality	0.186	0.162	0.006**
Family	Physical function 1	0.349	0.330	0.001**
Mobility	Physical limitation	0.255	0.261	0.001**
Self-care	Physical function	0.393	0.419	0.001**
Social	Social function	0.578	0.571	0.001**
Mood	Mental health	0.365	0.369	0.001**
Personality	Mental health	0.273	0.271	0.001**
Work	Physical limitation	0.145	0.151	0.032*

The SSQoL was interpreted in the Tamil language. There were no significant disparities in the interpretation, as the questionnaire failed to contain elements that could differ immensely among diverse cultures. Additionally, both patients and healthcare professionals found all questions and responses to be suitable and easy to understand without any modifications needed. Additionally, reliability and validity were assessed, ensuring a thorough evaluation.

Test-retest analysis results were highly significant with ICC values are more than 0.9 and Cronbach alpha values of more than 0.9 (Table 3). There was significant convergent validity with Spearman rho scores of 0.145 to 0.578 with a p-value less than 0.05 (Table 4). 

## Discussion

The main goal of the present analysis was to translate the SSQoL from English to Tamil among the Tamil-speaking population. This effort was particularly important as it corresponds with the worldwide practice of adapting valid and reliable clinical evaluation instruments for various linguistic and cultural communities, aiming to enhance patient care through evidence-based methods.

SSQoL-T questionnaire was designed to assess and categorize patients with chronic stroke survivors, based on their risk of poor prognosis in physical therapy, considering psychosocial factors. While the original SSQoL had been converted into several languages, including Spanish, French, Danish, Arabic, Dutch, German, Italian, Polish, Norwegian, Mandarin, Japanese, Swedish, Turkish, and Welsh, there had been no adaptation specifically for the large Tamil-speaking population [18-21,23,24].

Our findings revealed important insights into the demographic characteristics, convergent validity, and test-retest reliability of the SSQoL-T among patients with chronic stroke survivors. In the convergent validity analysis, Spearman's rank correlation coefficients (ρ) between the SSQoL-T and the original English version of SSQoL demonstrate strong positive relationships for both overall score and sub-score assessments. The test-retest reliability analysis revealed moderate to good reliability for both total-score and sub-score assessments of the SSQoL. The ICC values indicate reasonable consistency and stability over time, with ICC values of 0.986 for the total score and more than 0.980 for the sub-scores. ICC for the total and sub-scores falls within the range of good reliability. The convergent validity with all sub-scores between SSQoL-T and SF-36 showed a great correlation with p≤0.01, CI of 99%.

Overall, the results indicated that SSQoL-T was a liable and valid screening instrument for evaluating chronic stroke survivors among Tamil-speaking patients. These outcomes are crucial for clinicians and researchers working in the domain of stroke management, as they demonstrate the utility of the SSQoL in accurately identifying individuals at risk of poor prognosis and facilitating targeted to improve patient outcomes in the Tamil-speaking population [25-29].

Using the SSQoL-T can influence the type of care subjects with chronic stroke survivors in primary care settings. Those identified as high risk often face challenges in their recovery due to psychological factors, which may constrain their approach to it. It can be used to evaluate the abilities and problems of the individual and may also serve as a basis for intervention and management planning within the Indian context of return-to-work and sustaining.

Limitations

A homogenous group of samples could not be taken. It was difficult to interpret the results of cross-cultural adaptation with variations in slang, pronunciation, and interpretation of meaning in the Tamil language. It was very difficult to generalize the concepts in this original SSQoL questionnaire because of Tamil cultural variations in different sets of samples taken for this study.

## Conclusions

The reliable, valid original SSQoL English version was adapted and translated successfully to the South Indian language Tamil SSQoL-T in this study, demonstrating good psychometric properties. The adaptation process maintained the original tool's essence and integrity while ensuring its suitability for the Tamil-speaking population. Affected quality of life is the major concern and it is the negative consequence of chronic stroke survivors. SSQoL-T scale is a reliable, valid tool that has reasonable properties related to psychometrics and good convergent validity. SSQoL-T may be an appropriate instrument for use in future research in clinical and epidemiological studies among Tamil-speaking survivors of chronic stroke. Further, it is recommended that SSQoL be translated and validated in various Indian languages to get significant responses related to QoL in survivors of chronic stroke.
